# Intensive care for cancer patients: An interdisciplinary challenge for cancer specialists and intensive care physicians

**DOI:** 10.1007/s12254-016-0256-6

**Published:** 2016-03-08

**Authors:** Peter Schellongowski, Michael Kiehl, Matthias Kochanek, Thomas Staudinger, Gernot Beutel, On Behalf of Intensive Care in Hematologic-Oncologic Patients (iCHOP)

**Affiliations:** Department of Medicine I, Medical University of Vienna, Waehringer Guertel 18–20, 1090 Vienna, Austria; Department of Internal Medicine I, Klinikum Frankfurt Oder, Frankfurt, Germany; Department of Internal Medicine I, University of Cologne, Cologne, Germany; Department of Hematology, Hemostasis, Oncology, and Stem Cell Transplantation, Hannover Medical School, Hannover, Germany; http://www.ichop.eu/

**Keywords:** Cancer, Intensive Care Unit, Prognosis, Admission Criteria, Respiratory Failure

## Abstract

Every sixth to eighth European intensive care unit patient suffers from an underlying malignant disease. A large proportion of these patients present with cancer-related complications. This review explains why the prognosis of critically ill cancer patients has improved substantially over the last decades and which risk factors are of prognostic importance. Furthermore, the main reasons for intensive care unit admission – acute respiratory failure and septic complications – are discussed with regard to diagnostic and therapeutic specifics. In addition, we discuss potential intensive care unit admission criteria with respect to cancer prognosis. The successful management of critically ill cancer patients requires a close collaboration of intensivists with hematologists, oncologists and colleagues from other disciplines, such as infectious disease specialists, microbiologists, radiologists, surgeons, pharmacists, and others.

## Introduction

Only a few years ago, intensive care unit (ICU) mortality of critically ill cancer patients was unacceptably high, especially in those requiring invasive mechanical ventilation. Meanwhile, evidence-based intensive care unit admission criteria, general improvements in the management of organ dysfunctions, advances in the diagnosis and treatment of specific complications, as well as new therapeutic options for cancer and infections have led to a marked improvement of outcomes. The available data suggest that ICU survivors regain favorable quality-of-life, return to a state in which the continuation of anticancer therapy is feasible, and that their long-term survival as well as their hematologic and oncologic outcome may not be different from cancer patients who were never admitted to the ICU. Thus, a general reluctance to admit critically ill cancer patients to the ICU cannot be justified anymore [1–4].

The following article covers specific aspects of nonsurgical cancer patients admitted to the ICU. The acute respiratory failure (ARF) depicts by far the most common reason for an ICU admission in these patients, followed by septic complications and other partially cancer-specific conditions and emergencies.

## Respiratory insufficiency

The ARF is the most common reason for ICU admissions in critically ill cancer patients. The incidence of ARF ranges from 10 to 50 % in patients with hematologic or oncologic malignancies and goes as high as almost 90 % in some reports on allogeneic stem cell recipients [5]. Furthermore, it represents one of the most important risk factors for higher morbidity and mortality, especially, if invasive mechanical ventilation (IMV) becomes necessary. In addition, several diagnostic and therapeutic considerations apply. Thus, ARF depicts *the* central organ dysfunction in cancer patients.

### Prognostic importance of invasive mechanical ventilation

Until the 1980s, mortality rates of invasively ventilated cancer patients with ARF were up to 90 %. Over time, however, mortality rates have decreased markedly, even in cancer patients with ARF and IMV in addition to multiple organ failure and/or sepsis. A recent multicenter trial reports a mortality rate of only 52 % in hematologic patients with the most severe form of respiratory failure, the acute respiratory distress syndrome (ARDS). This exceeds mortality rates of the general ARDS population by only about 10 % [6]. Such advances can be attributed to improved patient selection, general progress of ventilatory strategies including concomitant therapies, increased understanding of adequate diagnostic measures (see below), as well as new antimicrobial substances, most of all antimycotics.

### Definitions, causes and diagnostics

The most common presentation of respiratory failure in critically ill cancer patients is hypoxic ARF (PaO_2_/FiO_2_-ratio < 200). Especially in hematologic patients, so-called respiratory events predict emerging oxygenation disturbances and imminent failure of the respiratory system: infiltrates, increased respiratory rates, cough, sputum, rales, thoracic pain, and hemoptysis are associated with increased intubation and mortality rates [7].

The prognosis of cancer patients is worse if the etiology of the ARF remains unclear [8–10]. A labor-intensive and evidence based workup of the multiple causes is associated with a diagnostic rate of approximately 80 % (see Table 1). In addition to noninvasive testing, bronchoalveolar lavage can increase the rate of positive findings in up to 20 % of cases and may be safely operated even in nonintubated patients, if a peripheral oxygen saturation of > 90 % can be obtained [11].

**Table 1:** Noninvasive tests in acute respiratory failure of cancer patients (adopted from [10])

Imaging studies	Chest radiograph
	High-resolution computed tomography
Echocardiography	Exclusion of pulmonary congestion
Sputum examination	Bacteria
	Fungi
	Tuberculosis
Induced sputum	P. jirovecii
Nasopharyngeal aspirates	Respiratory viruses
Blood cultures	
Polymerase chain reaction test	Herpes viridae
	Cytomegalovirus
Circulating Galactomanan	Aspergillus
Serologic tests	Chlamydia pneumoniae
	Mycoplasma pneumoniae
	Legionella pneumophila
Urine antigen	Legionella pneumophila
	Streptococcus pneumoniae

### Noninvasive ventilation as measure to avoid intubation?

The available literature of the past suggested the safety and efficacy of noninvasive ventilation (NIV) strategies as a measure to avoid intubation and mortality in immunocompromised patients. However, apart from some inconsistent observational trials, the evidence was based on two small and meanwhile historical randomized controlled trials (RCTs) including heterogeneous patient populations [8, 12]. The mortality rates in the respective control groups (O_2_-insufflation only) of these trials were excessive compared to recent studies. Thus, the findings of these investigations may have now lost relevancy. Moreover, several of the mentioned observational trials raised concerns that secondary intubation after NIV failure may be associated with even higher mortality rates when compared to primary IMV [8, 10, 13].

Very recently, one large prospective multicenter RCT and one large multicenter observational study with propensity score matching suggested that in specialized centers using prespecified ICU admission criteria and rigorous diagnostic testing for the etiology of ARF, the use of early NIV does not seem to be superior with regard to intubation rates and mortality when compared to O_2_-insufflation alone [14, 15]. On the other hand, both studies did not show any drawbacks associated with NIV. With regards to the RCT, it has to be stated that the mortality rate in the control group was lower than expected, so that the trial was underpowered. In addition, patient inclusion criteria seems to have been more liberal than in the historical trials. Eventually, the use of high-flow nasal cannula oxygen therapy, a novel and possibly promising therapy in patients with hypoxic ARF, was used in both arms of the study, which may itself have had an impact on the outcome [16].

Thus, even though the use of early NIV does not seem to be supported by recent evidence, the same data do not entirely rule out a role for NIV in certain situations. We suggest that if NIV is used in cancer patients with hypoxic ARF, close monitoring for established risk factors for NIV-failure and awareness for early break-off followed by endotracheal intubation is warranted (see Table 2 for detailed information on risk factors for NIV failure) [17].

**Table 2:** Risk factors for NIV failure in cancer patients with hypoxic ARF (adopted from [17])

Risk factors for NIV failure in cancer patients with hypoxic ARF	
Prior to NIV	Vasopressor need
	Multiple organ failure
	Airway involvement by malignancy
	Acute respiratory distress syndrome
	Unknown etiology for ARF
	Delayed onset of ARF
During NIV	Patient not tolerating NIV
	No improvement of ABG within 6 hours
	Respiratory rate > 30/minute
	NIV dependency ≥ 3 days
	Clinical or respiratory deterioration
	Unknown etiology for ARF

*NIV* noninvasive ventilation, *ARF* acute respiratory failure, *ABG* arterial blood gas

#### Sepsis

The risk of cancer patients for septic complications is increased up to ten times when compared to noncancer patients [18]. Cancer patients were excluded from participation in many RCTs on sepsis of the past. However, several subsequent observational studies could show that (1) suggested strategies derived from these RCTs were implemented also in cancer patients, (2) sepsis-associated mortality rates decreased markedly also in cancer patients, and (3) sepsis-associated mortality rates may not be different from mortality rates of sepsis patients without malignant diseases [19–21]. In fact, highly specialized centers report hospital mortality rates of 43 % in neutropenic patients with severe sepsis or septic shock in more recent years [19].

Nearly every second ICU cancer patient with septic complications has recently received chemotherapy and/or is presenting with neutropenia. Both factors do *not* have an impact on mortality [20, 22]. In neutropenic sepsis patients, favorable outcomes are associated with the removal of central venous lines in the absence of another infection focus, the identification of a sepsis-causing microorganism, as well as with an antibiotic regimen combining an anti-pseudomonas broad spectrum ß‑lactam with an aminoglycoside [19, 23]. In contrast, neutropenic sepsis is associated with increased mortality rates in case of higher degree of acute organ dysfunctions, need for IMV, as well as pulmonary or fungal infection [19, 20].

In neutropenic sepsis, antimicrobial therapy corresponds to the treatment of neutropenic fever, unless the prior antimicrobial prophylaxis and/or therapy, or a manifest or suspected focus longs for modification, e. g. in case of pulmonary infiltrates (such as mold-active systemic antifungal therapy in lung infiltrates not typical for Pneumocystis pneumonia or lobar bacterial pneumonia, high-dose trimethoprim**-**sulfamethoxazole in infiltrates compatible with P. jirovecii) [25]. Several guidelines on the use of antimicrobials in neutropenic cancer patients with infection are available to support clinical decision-making [24, 25]. Even though using G‑CSF formulations in neutropenic sepsis is not uncommon, there is no evidence for its efficacy and the use is not supported by guidelines [24, 26]. In contrast, observational studies point out that in case of pulmonary infection, respiratory impairment may be aggravated by G‑CSF-associated neutropenia recovery, supposedly due to local inflammation and increased lung microvascular permeability [27, 28].

## Other important conditions

Regardless of the reason for ICU admission, approximately 25 % of cancer patients present with at least one cancer-specific complication [19]. The management of drug reactions to immuno- and/or chemotherapy, hyperleukocytosis, tumor lysis syndrome, malignancy-related airway obstruction, stem cell transplant-associated conditions, increased occurrences of certain electrolyte disturbances, such as hypercalcemia or SIADH, thrombotic microangiopathies, as well as thrombotic or bleeding diathesis warrant for close collaboration of intensivists with hemato/oncologists and colleagues from other disciplines.

## Possible admission criteria

Any suggested admission criteria can only offer a gross orientation and must be evaluated on a case-by-case basis with regard to its applicability. Based on the existing evidence, the following approach seems to be reasonable [29]:

- A “full code management” without restrictions is advisable in patients in remission of their malignant disease, newly diagnosed malignancies with favorable life expectation (> 1 year), availability of curative therapeutic options (e. g. hematologic malignancies during induction or consolidation therapy), complications of autologous stem cell transplantation, in certain cases of low grade hematologic malignancies, in multiple myeloma with partial remission, as well as in patients with advanced stages of solid malignancies, if available therapeutic options still allow for long-term survival.
- The term “ICU trial” describes an initial “full code management” for three to five days followed by a thorough re-evaluation of the therapeutic strategy. This approach seems to be adequate in patients who do not fulfill the above stated criteria, but for whom the option of a potentially life-extending therapy is available. In patients of this category with at least two organ failure including IMV, Lecuyer et al. showed that no clinical sign at ICU admission correlates with hospital outcome [30]. Only *after* the third ICU day the severity level of organ dysfunction differed between survivors and nonsurvivors. The mortality in these very ill patients was as high as 80 %. No patient requiring an additional intensive care therapy measure (intubation, renal replacement therapy, vasopressors) after the third ICU day survived.
- According to the available literature, patients with no life-extending therapy option for their underlying malignant disease, with uncontrolled or refractory graft-versus-host-disease after allogeneic stem cell transplantation, unfavorable cancer-related life-expectation (< 1 year), and patients who were bedridden most of the time within the last three months should not receive aggressive ICU therapies. However, ICU admissions for management of a specific acute problem together with primary therapy limitations (such as do-not-intubate orders) may be suitable in selected patients [31].

## Unanswered questions

Several questions in the context of critically ill cancer patients remain unanswered:

- How effective is communication and collaboration between intensivists and hemato/oncologists with regard to clinical decision-making?
- What is the impact on outcome if no hemato/oncologist is available due to the institutional structure?
- What are the specific needs of critically ill cancer patients’ families?
- How can we optimize the transition from full code ICU management to palliative care, if indicated?
- Early ICU admissions seem to be associated with a favorable outcome in cancer patients with ARF. However, if this applies to other organ dysfunctions remains unclear. Moreover, a recent publication suggested that treating patients with acute leukemia with hematological risk of early death in the ICU even in the absence of a manifest organ failure is associated with improved survival [32].
- How can we establish effective structures to identify patients in the wards who would profit from ICU admission?
- How safe and effective is the administration of intensive care measures in the setting of a normal ward?
- What would be the benefits of hemato/oncologic intermediate care units?

## Volume dependency

The mortality rates of cancer patients with ARF and septic complications correlate with the number of treated cases per year per respective ICU *(*volume dependency) [21, 33]. So-called high-volume ICUs are more likely to be part of a tertiary care or university hospital with an affiliated department of hemato/oncology [21]. This fact needs to be considered whenever assessing the research results related to the subject, as these are usually derived from highly specialized centers. Even though various data reported from nonspecialized centers are promising, some authors still report on disproportionally high mortality rates [21, 33–35]. Given the fact that every sixth to eighth European ICU patient suffers from an underlying malignant disease, educational measures should be encouraged to ensure optimal treatment of these patients in case of critical illness [2, 36]. In complex cases, however, transfer of patients to an expert center should be evaluated.

The authors of this review suggest establishing local structures including joint educational sessions on the recent advances of cancer treatments and intensive care medicine, ICU admission policies for orientation, joint evaluation of ICU transfer candidates, daily interdisciplinary rounds of critically ill ICU cancer patients, as well as debriefing sessions subsequent to the treatment of challenging cases. Eventually, including basic elements of intensive care, as well as specific aspects of hematologic and oncologic patients into the curricula of future cancer specialists and intensivists, will benefit the patients [36].
